# Metaplastic Paneth Cells in Extra-Intestinal Mucosal Niche Indicate a Link to Microbiome and Inflammation

**DOI:** 10.3389/fphys.2020.00280

**Published:** 2020-03-31

**Authors:** Rajbir Singh, Iyshwarya Balasubramanian, Lanjing Zhang, Nan Gao

**Affiliations:** ^1^Department of Biological Sciences, Rutgers University, Newark, NJ, United States; ^2^Department of Chemical Biology, Ernest Mario School of Pharmacy, Rutgers University, Piscataway, NJ, United States; ^3^Rutgers Cancer Institute of New Jersey, New Brunswick, NJ, United States; ^4^Department of Pathology, Princeton Medical Center, Plainsboro, NJ, United States

**Keywords:** Paneth cells, Paneth cell metaplasia, microbiome, intestinal metaplasia, gastritis, Barrett’s esophagus

## Abstract

Paneth cells are residents of the intestinal epithelium. Abnormal appearance of Paneth cells has been widely documented in non-intestinal tissues within the digestive tract and even observed in non-gastrointestinal organs. Although metaplastic Paneth cells are part of the overarching pathology of intestinal metaplasia (IM), only a fraction of intestinal metaplastic lesions contain Paneth cells. We survey literature documenting metaplastic Paneth cells to gain insights into mechanism underlying their etiologic development as well as their potential relevance to human health. A synthesized view from this study suggests that the emergence of metaplastic Paneth cells at extra-intestinal mucosal sites likely represents a protective, anti-bacterial, and inflammatory response evoked by an altered microbial activity.

## Introduction

Paneth cells are a group of mature intestinal epithelial cells present in humans and other mammals. They are primarily localized at the base of the crypts of Lieberkühn in the small intestine. These cells were first described by Schwalbe (1872) and later fully characterized by Josef Paneth (1888). Normal Paneth cells differentiate from intestinal stem cells and live for about 60 days (Ireland et al., 2005). Paneth cells can be found in human cecum and ascending colon but are extremely rare in human distal colon and are absent in rodent colonic epithelium (Tanaka et al., 2001). Conventional histological examination identifies Paneth cells as large columnar epithelial cells with characteristic eosinophilic secretory granules densely packed in the cytoplasm. In addition to secretion of growth factors (e.g., Wnt and EGF ligands) (Clevers and Bevins, 2013) to maintain the intestinal stem cell niche (Clevers and Bevins, 2013), Paneth cells secrete numerous antimicrobial peptides (e.g., α-defensins, lysozyme, Reg3A, etc.) (Bevins and Salzman, 2011) to regulate mucosal immune response.

Loss or reduction of Paneth cells are found in ileal Crohn’s disease (Wehkamp et al., 2005; Perminow et al., 2010; Adolph et al., 2013), intestinal ischemia (Grootjans et al., 2011), necrotizing enterocolitis (McElroy et al., 2013), pathogenic bacterial infection (Zhang et al., 2012; Conway et al., 2013; White et al., 2017), and graft vs. host disease (Fishbein et al., 2008; Levine et al., 2013; Kroemer et al., 2016). In contrast, abnormal appearance of Paneth cells in other parts of the gastrointestinal and extra-gastrointestinal region is referred to as Paneth cell metaplasia (PCM). Abundant appearance of Paneth cells in the human distal colons have been extensively documented in literatures published on ulcerative colitis (Tanaka et al., 2001; Bedini et al., 2014; Simmonds et al., 2014), Crohn’s disease (Tanaka et al., 2001; Simmonds et al., 2014), and colonic tubular adenoma (Symonds, 1974; Wada et al., 2005; Shi, 2007; Wehkamp and Stange, 2010; Wang et al., 2011a; Mahon et al., 2016). Although these Paneth cell alterations are frequently associated with chronic inflammation, the molecular mechanism and significance of Paneth cell-related pathologies are poorly understood.

Intestinal metaplasia (IM) represents a gastrointestinal pathological condition and is defined as an abnormal presence of intestinal epithelial cells in non-intestinal tissues. Although not all IM contain Paneth cells, metaplastic Paneth cells have been widely reported in diseased upper alimentary tissues such as Barrett’s esophagus (BE) (Chen et al., 2015), chronic gastritis (Montero and Loizaga, 1971; Lewin et al., 1976; Rubio et al., 1987; Deveci and Deveci, 2004; Shen et al., 2005), and Brunner’s gland (Coutinho et al., 1996). Surprisingly, rare metaplastic Paneth cells were even observed in other gastrointestinal and non-gastrointestinal tissues (Symonds, 1974; Tanaka et al., 2001; Mitsuhashi et al., 2005; Puiman et al., 2011; Gassler, 2017). In addition to briefly summarizing the well-studied PCM in the left colon, which represents a hallmark change in idiopathic inflammatory bowel disease (IBD), this review will analyze literature on metaplastic Paneth cells in upper gastrointestinal or non-gastrointestinal tissues, an area of research that has received little attention. We noted a potential correlation between bacterial activity and the etiologic development of metaplastic Paneth cells within these extra-intestinal mucosal niche.

### Metaplastic Paneth Cells in Colon and Its Disease Relevance

Paneth cells are normally present in proximal (right and transverse) colons in human but are extremely rare in the distal (left) colon. Detection of Paneth cells in the left colon were referred to as PCM, and documented in many IBD studies (Lewin, 1969; Bansal et al., 1984; Ajioka et al., 2005; Simmonds et al., 2014). In addition, PCM in other colonic inflammation conditions such as diverticulitis (Sandow and Whitehead, 1979) and radiation colitis (Watanabe, 1978) had also been reported. However, the histogenesis of colonic PCM is not fully understood. Metaplastic Paneth cells in IBD colon were usually found in crypt regions and morphologically identical to normal Paneth cells that reside in the small intestines (Cunliffe et al., 2001). Immuno-histochemical studies indicated that colonic metaplastic Paneth cells expressed antimicrobial peptides: lysozyme (Klockars et al., 1977), sPLA2 (Haapamaki et al., 1997), and α-defensins (Cunliffe et al., 2001). Normal colonic mucosa does not express human alpha defensin 5 (HD5; antimicrobial protein produced by Paneth cells). However, HD5 was detected in colonic crypts of IBD patients’ samples, consistent with the occurrence of PCM (Cunliffe et al., 2001).

By reporting significantly higher PCM incidences in the distal colon of IBD patients compared to non-IBD and control patients, Tanaka et al. (2001) proposed an association of colonic Paneth cells with IBD. Regression analysis suggested that repair and regeneration might be the most potent stimuli causing PCM (Tanaka et al., 2001). Moreover, Paneth cells can sense commensal microbiota (Vaishnava et al., 2008) and shape the microbiome (Salzman et al., 2010) to maintain the intestinal homeostasis. Therefore, the presence of PCM could be an adaptive response to protect the damaged colonic epithelium against bacterial invasion. Additionally, Wehkamp et al. (2007) suggested that the antibacterial peptides produced by Paneth cells might counteract bacterial attack as an “on-demand” mechanism.

The detection of Paneth cells in colorectal adenomas was reported as early as 1967 (Gibbs, 1967) and the reported frequencies of such detection varied from 0.2 to 39% in different studies (Bansal et al., 1984; Wada et al., 1994, 2005; Joo et al., 2009; Pai et al., 2013; Mahon et al., 2016). Furthermore, Paneth cells not only produce antimicrobial components but also constitute an important epithelial niche for small intestinal stem cells by producing epithelial growth factor (EGF), transforming growth factor (TGF-α), Wnt3, and Notch ligands (Sato et al., 2011). The IBD patients have an increased risk to develop inflammation-associated colon cancer. Additionally, during repair and regeneration of damaged epithelium in IBD patients, there might be an increased chance of mutation accumulation (cyclic hit model, described in later paragraphs), and the metaplastic Paneth cells may contribute to accelerated epithelial tumorigenesis by providing stem cell growth factor to tumor cells (Chen and Huang, 2014).

### Metaplastic Paneth Cells in Gastric Mucosa and Its Disease Relevance

Intestinal metaplasia in human stomach was first reported by Morson (1955) and later recognized as a precursor for gastric cancer in various studies (Morson, 1955; Ming et al., 1967; Matsukura et al., 1979; Segura and Montero, 1983; Shimada et al., 1987). Kawachi et al. (1976) used Tes Tape method to characterize IM by disaccharidase visualization and classified the lesions into two types. In type I IM, also known as the complete IM, goblet cells, Paneth cells, as well as enterocyte enzymes, including sucrase, maltase, trehalase and alkaline phosphatase were present. In type II, the incomplete form of IM, trehalase, alkaline phosphatase, and Paneth cells were absent. Thus, complete metaplasia partially resembles small intestinal epithelium containing Paneth cells whereas incomplete metaplasia resembles colonic epithelium lacking Paneth cells. In their study, out of 96 samples of IM, 57% showed type I IM with the presence of Paneth cells, while 43% were type II lacking Paneth cells (Kawachi et al., 1976).

By using various staining procedures (Table 1), Albedi et al. (1984) reported that immature Paneth cells were present in less differentiated IM. Other authors also confirmed the presence of PCM by immunostaining for PSTI (Pancreatic secretory trypsin inhibitor) (Kazal et al., 1948; Bohe et al., 1986, 1987), lysozyme (Heitz and Wegmann, 1980), and human defensin 5 (Shen et al., 2005). Additionally, Paneth-like cells were reported in gastric adenomas and carcinomas (Heitz and Wegmann, 1980; Ito et al., 1986; Caruso et al., 1989; Rubio, 1989). Although some of these tumors exhibited aggressiveness (Ohtani and Sasano, 1988), the prognostic value of these Paneth-like cells for gastric carcinoma remain unclear.

**TABLE 1:** Methods used for identification of metaplastic Paneth cells.

Organ	Method of identification	References
Esophagus	Conventional H&E staining	Chen et al., 2015
	H&E, light microscopy and electron microscopy	Schreiber et al., 1978
	H&E, periodic acid-Schiff/Alcian blue	Takubo et al., 1995
	H&E, Lendrum’s Phyloxine-tartrazine stain	Thompson et al., 1983
Stomach	Periodic acid-Schiff/Alcian blue at pH 2.5	Abraham et al., 2003
	H&E, Masson’s Trichome, Lendrum’s Phyloxine-tartrazine, Periodic acid-Schiff/Alcian blue at pH 2.5 and colloidal iron pH 1.9 staining	Albedi et al., 1984
	H&E, Periodic acid-Schiff/Alcian blue at pH 2.5, immunostaining for lysozyme	Deveci and Deveci, 2004
	Immunostaining for lysozyme	Gassler et al., 2002
	Conventional H&E staining	Inada et al., 1997
	H&E, Periodic acid-Schiff/Alcian blue at pH 2.5 and Electron microscopy	Matsubara, 1977
	Immunoelectron microscopy for lysozyme	Ohtani and Sasano, 1988
	Immunostaining for HD5 and HD6	Ostaff et al., 2015
	Conventional H&E staining	Rubio et al., 1987
	Lysozyme immunostaining	Rubio, 2012
Gallbladder	Conventional H&E staining	Albores-Saavedra et al., 1986
	Conventional H&E staining	Bae et al., 2002
	Electron microscopy, H&E, Periodic acid-Schiff	Laitio and Nevalainen, 1975
	Conventional H&E staining	Sakaki et al., 2000
Ovary	Conventional H&E staining	Alrajban et al., 2018
	Conventional H&E staining	Niemiec et al., 1989
Prostate	Conventional H&E staining, immunostaining for lysozyme	Frydman et al., 1992
	Conventional H&E staining	Adlakha and Bostwick, 1994

Inada et al. (1997) further classified gastric IM on the basis of differentiation status into two major classes (i) gastric and intestinal (GI) mixed type; and (ii) solely intestinal (I) type. Six subclasses were further established for the mixed GI type. In this study, they observed the presence and absence of Paneth cells in IM at almost the same incidence rate i.e., 15 and 17%, respectively in fundic mucosa. The incidences of intestinal subtypes with Paneth cells were 17% in fundic and 19% in pyloric mucosa, respectively. The incidences of GI(Su-Pa+) subtypes which are characterized as showing presence of Paneth cells (Pa) and absence of surface mucous cells (Su) were 11% in fundic and 14% in pyloric mucosa (Inada et al., 1997). Within these metaplastic lesions, the presence of Paneth cells near the proliferative zone and the large intracellular granules suggested that these cells were neither chief cells nor pyloric gland cells (Inada et al., 1997).

Matsubara performed morphological characterizations of Paneth cells and reported that Paneth cells in normal small intestinal epithelia contained more phagolysosomes in lower cytoplasm compared to Paneth cells found in IM (Matsubara, 1977). This represented one of the earliest attempts to distinguish intestinal resident cells vs. metaplastic Paneth cells. Gassler et al. (2002) later reported that the expression of calnexin was inversely related to secretory lysozyme in Paneth cells of normal intestinal mucosa but became directly correlated to lysozyme in metaplastic Paneth cells in gastric mucosa. They further identified the expression of proliferative marker Ki67 in metaplastic Paneth cells but not in Paneth cells of normal small intestines. These studies suggested that metaplastic Paneth cells may behave differently from normal Paneth cells (Gassler et al., 2002). However, detailed information at the molecular level is currently absent to distinguish normal from metaplastic Paneth cells.

Most human gastric cancers arise after long-term *Helicobacter pylori* infection via progression of metaplastic changes, first named by Correa (Correa, 1992; Correa and Shiao, 1994; Uemura et al., 2001) with the first metaplastic change that increases risk for progression to cancer being atrophic gastritis, involving loss of acid secreting parietal cells with concomitant pseudopyloric metaplasia (aka SPEM, discussed below) of the remaining cells. Some authors believe IM is a requisite step after atrophy (Correa’s hypothesis), whereas others like David Graham (Graham et al., 2019) have suggested IM is more of a reparative lesion, not directly related to carcinogenesis, and atrophy is the more diagnostic risk factor (El-Zimaity et al., 2002). In gastric atrophy, caused by long-term *H. pylori* colonization, mature chief cells that are present at the base of glands are replaced by (metaplastic) cell types that co-express both the chief cell marker and markers of normal mucus-secreting cells residing in the gland neck, such as Trefoil Factor 2, TFF2 (spasmolytic polypeptide). This type of metaplasia was also known as spasmolytic polypeptide-expressing metaplasia (SPEM) or pseudopyloric metaplasia due to the lack of mature chief and parietal cells, resembling antrum or pyloric epithelium (Goldenring et al., 2011; Saenz and Mills, 2018). During SPEM, the differentiated cells change their fate to a regenerative state by first shutting down the mTORC1 signaling followed by expression of progenitor markers to reactivate mTORC1. This process was named as paligenosis (Jin and Mills, 2018; Willet et al., 2018). In mouse models, SPEM emerged in chronically inflamed stomach and clearly proceeded to give rise to gastric cancer (Fox et al., 1996). Inflammation due to infection or other insults increases paligenotic events. This continuous process of paligenosis increases the risk of accumulation of mutations leading to emergence of a neoplastic or dysplastic clone. This phenomenon was called “cyclical hit model” (Burclaff and Mills, 2018; Saenz and Mills, 2018; Jin and Mills, 2019). Although there is no study that has explicitly studied how Paneth cells originated during the process of metaplasia, a recent study by Leushacke et al. (2017) showed LGR5+ subpopulation of chief cells co-expressed intestinal stem cell and Wnt markers in advanced intestinal type tumors in the corpus. Therefore, metaplastic Paneth cells could be part of the program of IM, the origin of which may relate to infection and inflammation but remain poorly understood.

Although the relevance of metaplastic Paneth cells during gastric mucosal pathogenesis are not clear, the characteristic production of antimicrobial peptides by Paneth cells suggested that the metaplasia might be an adaptive response to bacterial infection. Paneth cells secrete defensins and lysozyme shown to reduce the colonization of *H. pylori* in the stomach (Tanabe et al., 2008). Interestingly, mucosal areas with IM or with pseudo-pyloric metaplasia showed a lack of *H. pylori*, suggesting presence of Paneth cells in the metaplastic mucosa may be an adaptation against bacterial infection (Rubio, 2015). Gastric atrophy is characterized by the loss of chief and parietal cells and during infection this atrophy progresses from antrum to corpus (Kimura et al., 1996; Shichijo et al., 2015). As *H. pylori* infection is associated with IM and a higher risk of cancer, attempts were made to reduce the infection (Fukase et al., 2008; Ogura et al., 2008; Sakitani et al., 2011; Shichijo et al., 2016). Some studies reported that IM did not seem alleviated even after eradication of *H. pylori* infection, suggesting the potentially irreversible nature of IM (Wang et al., 2011b; Kodama et al., 2012; Mera et al., 2018). However, other studies showed that eradication of *H. pylori* somewhat reduced the IM (Correa et al., 2000; Zullo et al., 2000; Kong et al., 2014). In addition, Paneth cell antimicrobial peptides, especially human defensin 5 and 6, were recently found upregulated in gastric mucosa of heavy alcohol users (Ostaff et al., 2015). Thus, IM in gastric mucosa might also be caused by non-bacterial factors such as excessive alcohol use, retrograde bile reflux, aspirin, and anti-inflammatory drugs (Webb et al., 1996; Bresalier, 1998). However, these changes may also indirectly alter the gastric colonization of *H. pylori* or other bacterial species. More studies are required to elucidate these causal relationships.

### Metaplastic Paneth Cells in Esophageal Mucosa and Disease Relevance

The presence of Paneth cells in BE was first described by Schreiber et al. (1978), when metaplastic Paneth cells similar to intestinal resident Paneth cells were detected in BE biopsy samples from 4 patients. BE is characterized by a single layer of columnar epithelium replacing the native multi-layered stratified squamous epithelium of distal esophagus. BE is considered as a form of protective adaption from chronic insults, secondary to gastroesophageal reflux disease (Burgess et al., 1971; Naef et al., 1975; Ozzello et al., 1977), and the precursor to most esophageal adenocarcinomas. This esophageal disorder is pathologically manifested by the presence of multiple intestinal epithelial cell lineages including Paneth cells and enteroendocrine cells along with gastric cells (Boulton et al., 2003).

Later, Thompson et al. (1983) exhibited the presence of Paneth cells in 50% of the esophagogastrectomy samples of Barrett’s metaplasia and specimens with adenocarcinoma at the gastroesophageal junction. No difference in sex ratio or age range was observed among the samples associated with or without metaplasia. Compared to earlier studies using biopsy samples, sampling error was minimized in this study using esophagogastrectomy. Moreover, various experimental approaches including scanning electron microscopy, specimen radiography, and dissecting microscopy along with regular histochemical techniques were used in this study in comparison to earlier studies that mainly relied on histologic analysis.

In the last decade, immunostaining for various antimicrobial peptides was used to identify Paneth cells. Shen et al. (2005) reported human defensin 5 immunostaining as a tool for identification of IM in BE as well as in gastric IM. Under normal conditions, human defensin 5 is present in intestinal Paneth cells, and is generally confined to the small intestinal epithelium. However, the expression of defensin 5 was also observed in IM. The frequency of human defensin 5 staining was higher in gastric IM compared to BE. This difference was attributed to higher *H. pyloric* infection in stomach compared to esophagus (Shen et al., 2005).

Rubio also reported the presence of PCM in BE by detecting expression of lysozyme and other Paneth cell-specific antimicrobial proteins (Rubio, 2012). In a recent study, Chen et al. (2015) conducted a large study on 757 esophageal biopsy specimens, and reported that 31% of this cohort with IM contained metaplastic Paneth cells. These results were in accordance with previous reports showing a similar frequency of PCM in BE (Schreiber et al., 1978; Thompson et al., 1983; Takubo et al., 1995). Moreover, the highest incidence of PCM in BE was observed in indefinite dysplasia and low grade dysplasia samples. Additionally, in their follow-up study, they showed PCM was associated with reduced disease regression and suggested it as a potential marker for identification of severe disease (Chen et al., 2015). Moreover, presence of metaplastic Paneth cell products such as antimicrobial peptides may accelerate the cascade of BE by altering the expression of E-cadherin, thereby reducing the cell-cell interaction (Nomura et al., 2013).

Similar to gastric IM, BE was also viewed as an adaptation following chronic inflammation or injury to the esophageal epithelium. Bile acid was reported as one of the major contributor to the development of BE in animal models (Clark et al., 1994; Quante et al., 2012). Patients with gastroesophageal reflux disease often received proton pump inhibitors, which could cause reduced gastric acid secretion leading to bacterial overgrowth in BE. Additionally, it was reported that BE biopsies were highly associated with presence of bacteria compared to esophageal biopsies without BE (Osias et al., 2004). Two different types of microbiome were observed in esophageal biopsies: type I was dominated by genus *Streptococcus* and present with phenotypically normal esophagus; and type II contained Gram-negative anaerobes that were correlated with BE (Yang et al., 2009). Other studies also reported the presence of residential bacterial population in gastroesophageal reflux disease and BE. One study showed the presence of Bacteroidetes, Firmicutes, Proteobacteria, and Actinobacteria (Pei et al., 2005), while the other reported 46 bacterial species including high levels of *Campylobacter consisus* and *Campylobacter rectus* (Macfarlane et al., 2007). Liu et al. (2013) also observed Firmicutes, Proteobacteria, *Bacteroides*, *Fusobacterium*, and Actinobacter in 6 cases of BE. These studies suggested a potential rational toward a self defense mechanism against pathogenic bacteria. Therefore, presence of metaplastic Paneth-like cells in these lesions may represent an adaptive antibacterial response by the infected mucosa. However, besides an antibacterial role, defensins were also proposed to reduce E-cadherin in esophageal epithelium to possibly accelerate the BE pathogenesis (Nomura et al., 2013).

### Appearance of Paneth Cells in Other Gastrointestinal and Non-gastrointestinal Organs

Intestinal metaplasia has also been observed in several other gastrointestinal tissues. Although IM in gallbladder is very rare, complete IM was reported in gallbladder as early as 1967 (Jarvi and Lauren, 1967). Laitio and Nevalainen (1975) studied 100 gallbladders following cholecystectomies to remove gallstones. One specimen showed the presence of Paneth cells. Electron microscopy of this sample showed Paneth cells in columnar shape with apical cytoplasm occupied by secretory granules and well-developed Golgi apparatus. Later, Albores-Saavedra et al. (1986) reported that 3 out of 49 gallbladder samples showed the presence of Paneth cells. The presence of similar morphology in the IM in gallbladder to those in the stomach suggested that gastric and gallbladder epithelia have the same potential to differentiate into similar cell lineages. Although the IM of gallbladder containing Paneth cells was suspected to be associated with carcinoma development, the presence of higher immunoreactivity of lysozyme in non-dysplastic mucosa than in carcinoma did not support the hypothesis. Furthermore, the frequency of observing Paneth cells was found to be lowest in gallbladder cancer tissues compared to non-neoplastic mucosa, suggesting an inverse correlation of PCM to gallbladder cancer (Yamamoto et al., 1989). This observation was consistent with a recent comprehensive case-control study of 1,900 colorectal adenomas showing metaplastic Paneth cells in distal colorectal adenomas were inversely associated with synchronous advanced adenoma and carcinoma (Mahon et al., 2016). Sakaki et al. (2000) observed a case of gallbladder adenocarcinoma with extensive PCM.

Moreover, PCM was also observed in intestinal type cholangiocarcinoma associated with hepatolithiasis in large hepatic bile duct (Bae et al., 2002). Abnormal cholesterol and bile acid metabolism and secretion are common pathophysiological defects in gallbladder-related diseases. The presence of metaplastic Paneth cells in these diseases could be attributed to the presence of bacterial community. Bile-acid metabolism was reported to be regulated by the gut microbiome, thus the role of microbiota in disease cannot be overruled (Abeysuriya et al., 2008; Wang et al., 2009; Sayin et al., 2013). Similarly, the presence of microbiota in lithogenic bile could also promote inflammation and gall stones (White et al., 2006; Capoor et al., 2008; Sekirov et al., 2010) and microbiota association with gallstones was well reported (Kaufman et al., 1989; Lee et al., 1999; Maurer et al., 2005; Saltykova et al., 2016). Additionally, various microbes have also been isolated from bile in patients with cholesterol stones (Stewart et al., 1987; Cull and Beck, 1988; Darko and Archampong, 1994). Although, no study has established a direct correlation of PCM and the presence of bacteria in gallbladder related diseases, existing studies showed the presence of bacterial communities in lithogenic bile (Abeysuriya et al., 2008) and metaplastic Paneth cells in gallbladder with gallstones (Laitio and Nevalainen, 1975). Therefore, the presence of Paneth cells could be a protective mechanism against the pathogenic bacteria in lithogenic bile.

Alrajban et al. (2018) reported the first PCM in Krukenberg tumors, which represents 1–2% of ovarian tumors and are characterized by the presence of metastatic adenocarcinoma cells. A poorly differentiated carcinoma was observed to contain a mixture of signet ring malignant cells with eosinophilic cytoplasm characteristic of PCM. PCM was also observed in the lining of urethral diverticulum (Niemiec et al., 1989), a condition in which a variable sized pouch is formed. The *E. coli* was the most common organism among other enteric gram-negative bacteria isolated from urethral diverticulum patients (Ljungqvist et al., 2007; Greiman et al., 2019). Since urethral diverticulum is connected to the urethra and filled during urination, the metaplastic change in urethral lining could be due to exposure to irritants like bacterial colonization and retained urine. Thus, the metaplastic Paneth cells could again be an anti-bacterial response in this scenario. The reported presence of Paneth cells in neovaginitis (van der Sluis et al., 2016) might suggest a similar adaptive response to combat bacterial presence.

Intestinal metaplasia has been described in both benign and malignant prostate glands following estrogen therapy (Bainborough, 1952; Maung et al., 1988). Paneth cell-like metaplasia of prostate gland was first reported by Frydman et al. (1992). Adlakha and Bostwick later reported that these Paneth cell-like changes resembled intestinal Paneth cells by light microscopy. However, they lacked lysozyme immunoreactivity, and retained many prostate markers. Since these Paneth-like cells were positive for neuroendocrine markers, they were called “neuroendocrine cells with large eosinophilic granules” (Adlakha and Bostwick, 1994). PCM was also found in epididymis disorders (Nevalainen et al., 2001; Nistal et al., 2007). Together, these Paneth-like cells may represent transformed normal or neoplastic prostate cells.

The association of infection and inflammation had been investigated in many prostate cancer studies. In a recent study, Shrestha et al. (2018) showed that urine samples of patients with biopsy-proven prostate cancer had increased bacterial clusters frequently associated with other urogenital infections such as prostatitis and bacterial vaginosis (Massari et al., 2019). Additionally, a recent study testing prostatic fluid from prostate cancer and non-prostate cancer patients showed the presence of microbes in both samples, but revealed a difference in microbial species (Ma et al., 2019). Similarly, the presence of microbiota within tumoral, peritumoral and non-tumoral prostate tissue has also been reported (Cavarretta et al., 2017). Thus, whether emergence of Paneth like cells in prostate pathology represented another form of protective mechanism against bacteria may require future investigation.

## Closing Remarks

The mucosa of the gastrointestinal tract is continuously challenged by various micro-environmental factors ranging from pathogenic and opportunistic bacteria, and their products, to harsh secretions with digestive properties. In turn, human body has developed defense mechanisms like peristalsis, continuous revival of lining epithelium, and production of antimicrobial peptides, such as those secreted by the Paneth cells. The ectopic expression and secretion of these antimicrobial peptides in non-intestinal mucosa where they are normally absent illustrates a robust tissue plasticity in adaptation to infection and injury. Future studies are necessary to delineate the responsible microbial signaling pathways that invoke such unusual cellular metaplasia. The impact of these widely emerged metaplastic Paneth cells on the progression of inflammatory diseases and cancers warrants in-depth investigation such as using Paneth cell lineage tracing approaches (Yu et al., 2018).

## Author Contributions

RS, IB, LZ and NG conceptualized the manuscript. RS and NG drafted the manuscript. IB and LZ edited the manuscript.

## Conflict of Interest

The authors declare that the research was conducted in the absence of any commercial or financial relationships that could be construed as a potential conflict of interest.
